# The perception of Obstructive Sleep Apnoea/Hypopnoea Syndrome (OSAHS) among Italian general practitioners

**DOI:** 10.1186/s12948-015-0009-9

**Published:** 2015-04-15

**Authors:** Carlo Lombardi, Eleonora Musicco, Germano Bettoncelli, Manlio Milanese, Gianenrico Senna, Fulvio Braido, Giorgio Walter Canonica

**Affiliations:** Allergy and Pneumology Departmental Unit, Fondazione Poliambulanza Hospital, Brescia, Italy; General Practitioner, SIMG (Società Italiana di Medicina Generale), Brescia, Italy; Division of Pulmunology, S. Corona Hospital, Pietra Ligure, Italy; Allergy Service, Verona Major Hospital, Verona, Italy; Allergy and Respiratory Diseases, DIMI, University of Genoa, Genoa, Italy

**Keywords:** Obstructive Sleep Apnoea/ Hypopnoea Syndrome (OSAHS), General Practitioners (GPs), Polysomnography, Mechanical ventilation with positive airway pressure (C-PAP)

## Abstract

**Background:**

Obstructive Sleep Apnoea/Hypopnoea Syndrome (OSAHS) is a common disorder in the general population but often underestimated and underdiagnosed.

**Methods:**

This questionnaire-based study evaluated the overall level of knowledge about OSAHS among Italian General Practitioners (GPs), who are frequently involved in the management of this complex disease. This represents an interesting aspect, because GPs intercept many of the patients with OSAHS, in which C-PAP could be potentially indicated. Randomly-selected GPs were provided with questionnaires, which were then returned anonymously.

**Results:**

80 questionnaires have been validated; the participants in the sample examined were represented by 43 females and 37 males; the average age of participants was 51 years. The general knowledge on OSAHS is overall satisfactory among GPs; it is recognized by most of the GPs interviewed as pathology in constant increase, and associated with predisposing factors such as obesity. High blood pressure is perceived as an independent cardiovascular risk factor in patients with OSAHS, in line with the majority of international studies. The C-PAP has been identified as the care gold standard in patients with OSAHS, despite the lack of patient compliance in relation to this procedure, while polysomnography was found to be the main instrumental procedure used in the diagnostic workup of OSAS. The pulmonologist and a multidisciplinary team have been identified as the specialist figures of reference to which to direct the patient through the diagnostic workup. Respiratory therapists and nurses represent the role of educator in the proper management of the C-PAP in the opinion of 62% of respondents, while only 34% think that this role should be played by the GPs and/or other specialists.

**Conclusions:**

In conclusion, this survey about the perception of OSAHS among GPs in Italy highlighted a satisfactory overall knowledge of OSAHS and only few weak points.

## Background

OSAHS is a common disorder in the general population but often underestimated and underdiagnosed [1]. The definition of the Obstructive Sleep Apnoea/Hypopnoea Syndrome (OSAHS) cannot be separated from an integration of clinical and instrumental data. Clinically, OSAHS is characterized by excessive daytime sleepiness and/or alterations to the performance day and night snoring; from the pathophysiological point of view OSAHS is characterized by the occurrence during sleep of repeated episodes of partial or complete obstruction of the upper airways that are associated with oxygen concentration phasic reductions resulting in oxygen desaturation of arterial hemoglobin [2]. There are different degrees of obstruction of the upper airways that can determine the different respiratory events: complete obstruction (obstructive sleep apnea) and partial obstructions (divided into: hypopneas and Respiratory Effort Related Arousal (RERA). According to international standards for the diagnosis of OSAHS, the duration of respiratory events of each of the types described above shall not be less than 10 seconds and no more than 3 minutes. The relief of the obstructive events are processed using polygraphic instruments recording the activity of the subject during an entire night’s sleep [3]. The registration of an average number of complete obstructive respiratory events (apneas) and/or incomplete (hypopneas, RERA) per hour of sleep (RDI, Respiratory Disorder Index), equal to or > 10, configure a condition of OSAHS in adult subject. Several lines of evidence have identified OSAHS as an independent risk factor of secondary hypertension, such as obesity [4-6]. The treatment of OSAHS includes procedures such as mechanical ventilation with positive airway pressure (C-PAP), orthodontic devices, weight loss in obese subjects, positional therapy and surgery in those patients who have episodes of obstructive apnea sleeping supine. Some studies have shown that C-PAP improves daytime and nighttime blood pressure in patients with moderate and severe OSAHS, highlighting the importance of management and adherence to C-PAP therapy, in particular to normalize the oxygen concentration [7]. The present study evaluated the overall level of knowledge about OSAHS among italian General Practitioners (GPs), who are frequently involved in the management of this complex disease. This represents an interesting aspect, because GPs intercept many of the patients with chronic illness [8], as OSAHS, in which C-PAP could be potentially indicated.

## Methods

The results of this study were obtained through the completion of an anonymous questionnaire, created “*ad hoc”* and based on the guidelines and the current literature, by a panel of experts composed by General Practitioners (GPs) and Specialists (like Pneumologists, ENT specialists, and Allergologists). It consists of 16 items (Table 1) and is distributed to GPs to assess the level of knowledge of the clinical and therapeutical problems linked to OSAHS. Questionnaires were e-mailed to GPs over the entire territory of Brescia, a northern town of Italy, and had to be returned anonymously. Only the fully completed questionnaires were considered for the descriptive statistics. 80 questionnaires have been validated. The participants in the sample examined were represented by 43 females and 37 males; the age range of participants was : 34–65 years, and the average age was 51 years. Written informed consent was obtained for publication of this report.

**Table 1:** **“Questionnaire distributed to the participating Italian general practitioners (GPs)”**

**1. What is the definition commonly used for OSAHS?**	A) Daytime sleepiness associated with irregular breathing at night
	B) Episodes of paroxysmal dyspnea caused by obstruction of the upper and lower respiratory tract
	C) Obstructive Sleep apnea induced by gastroesophageal reflux
	D) Obstructive Sleep Apnea induced by persistent rhinitis characterized by nasal obstruction
	E) Obstructive Sleep Apnea induced by nocturnal asthmatic attack
**2. In the western countries, which is the males prevalence of OSAHS ?**	A) 2%
	B) 4%
	C) 8%
	D) 10%
**3. In the western countries, which is the females prevalence of OSAHS?**	A) 2%
	B) 4%
	C) 8%
	D) 10%
**4. Among your patients, how many have a diagnosis of OSAHS?**	A) No one
	B) 0–5
	C) 5–10
	D) >10
**5. The number of OSAHS patients in recent years has been:**	A) Stationary
	B) In growth
	C) In reduction
	D) Do not know
**6. OSAHS episodes of apnea/hypopnea can be of type:**	A) Obstructive
	B) Central
	C) Mixed
	D) All of the above
	E) Restrictive
**7. Is it true that obese people present a higher risk of developing OSAHS?**	A) Yes
	B) No
	C) Yes, but only women and children
	D) Yes, but also normal weight individuals can be affected
**8. What is the prevalence of OSAHS in the hypertensive patients?**	A) <5%
	B) Between 10 and 20%
	C) Between 20-40%
	D) > 50%
**9. Do you think that OSAHS should be considered an independent cardiovascular risk factor?**	A) Yes
	B) No
	C) I don’t know
**10. Are you aware of the questionnaire referred to as “Epworth Sleepiness Scale”?**	A) Yes, I use it in my professional activity
	B) Yes, but do not use it
	C) No, I do not know
**11. In the suspected diagnosis of OSAHS, what is the specialist that you turn more often in the first place?**	A) ENT Specialist
	B) Pulmonologist
	C) Allergist
	D) Internist
	E) Multidisciplinary Team
	F) Speech Therapist
	G) Psychologist/Psychiatrist
**12. When accessing the specialist’s assessment, which are the main problems you encounter?**	A) Waiting time
	B) Costs
	C) Communication with the specialist
	D) Degree of belief of the patient
**13. Which exam do you see as the most crucial for an accurate diagnosis of OSAHS ?**	A) Spirometry
	B) Maxilo-facial CT
	C) Determination of nocturnal oximetry in continuous
	D) Polysomnography
	E) EEG
	F) ECG Holter with integrated pressure arterial Holter
**14. What is the OSAHS treatment of choice?**	A) Nasal septum plastic intervention
	B) Uvulopalatopharyngoplasty (UPPP)
	C) Treatment with topical nasal steroids
	D) Topical association therapy with bronchial Long Acting Bronchodilators (LABAs) and steroids (iCSs)
	E) Nocturnal Oxygen continuous
	F) C-PAP
	G) Intraoral Orthodontic Devices
	H) Sleeping pharmacological agent (like benzodiazepines)
	I) Psychological approach
	L) Yoga
**15. When C-PAP is prescribed, patient reaction to the treatment is predominantly of :**	A) Total refusal
	B) Total adherence
	C) Lack of acceptance
**16. Who primarily has the responsibility of educating and supporting the patient in correcting and adjusting the C-PAP?”**	A) GP
	B) Specialist
	C) Nurse
	D) Respiratory Physiotherapist

## Results

**Figure 1:**
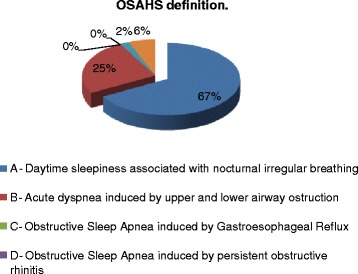
**“What is the definition commonly used for OSAS? “**

**Figure 2:**
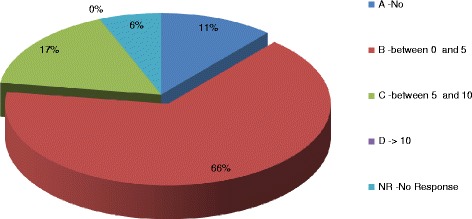
**“Among your patients, how many have a diagnosis of OSAHS?“**

**Figure 3:**
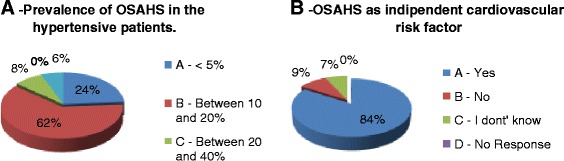
**“Answers to the question about the prevalence of OSAHS in hypertensive patients (3A) and about the perception of OSAHS as an independent cardiovascular risk factor (3B)”.**

**Figure 4:**
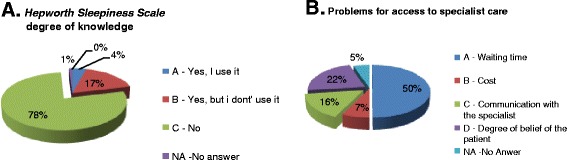
**GP's knowledge about the Hepworth questionnaire (A) and issues in access to specialist care (B).**

**Figure 5:**
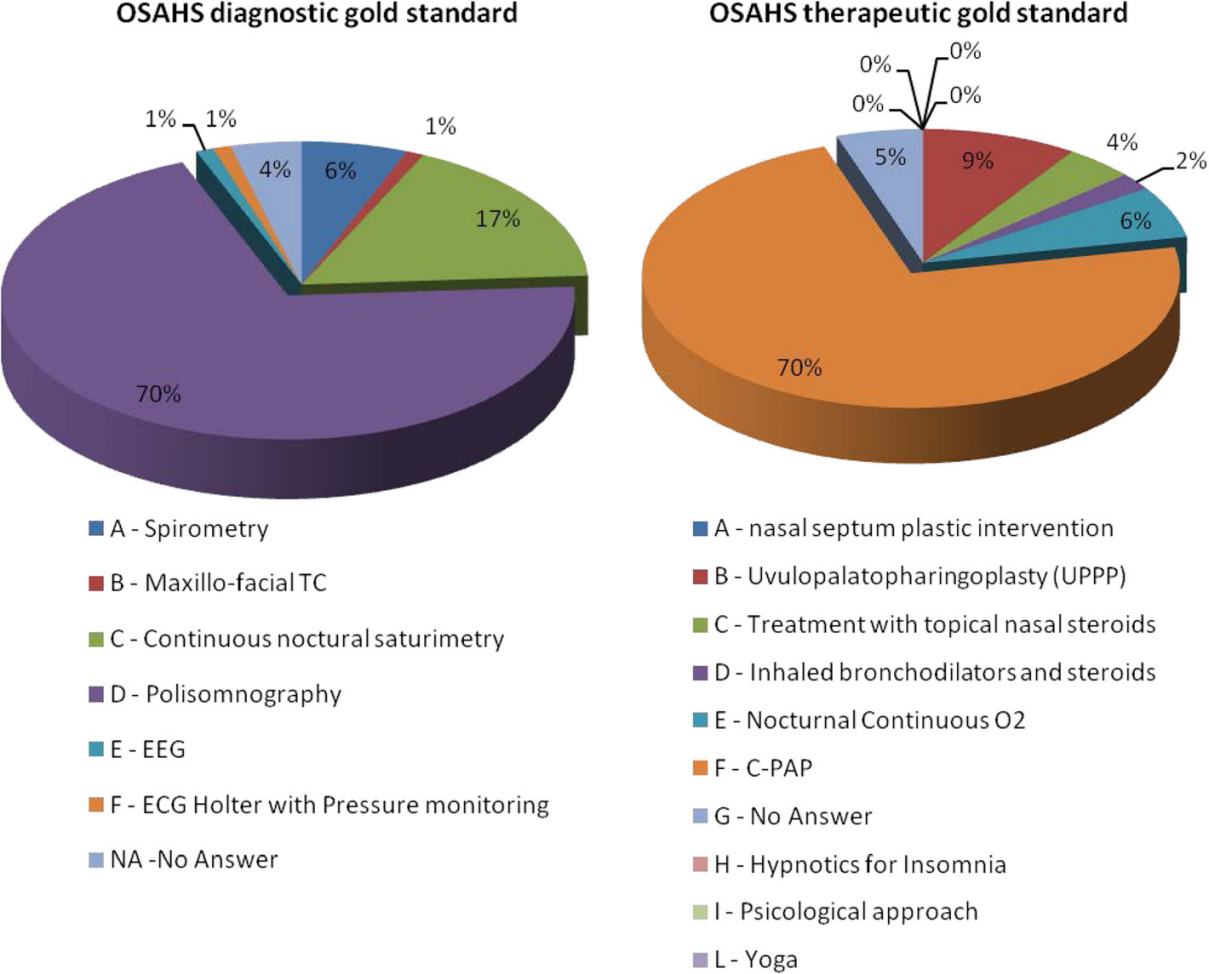
**“OSAHS diagnostic (A) and therapeutic (B) Gold standard“.**

As a general result, a good knowledge of the definition of OSAHS emerged from the results, with 67% of correct answer to the corresponding question in the questionnaire (Figure 1). An adequate level of knowledge is also found for the epidemiological data (Figure 2). OSAHS has a prevalence of 4% in males and 2% in female subjects aged between 30 and 60 years, 1% of preschool children and 11% in those aged > 61 years. More than half of the participants answered the question correctly on the prevalence of OSAHS in men (52% correct answers) and female (59% of correct answers). The prevalence of OSAHS among GPs surveyed is found to be low (0–0.5%) in line with the known under-diagnosis of OSAHS in the general population. The incidence of OSAS in the general population is 11% and is in constant increase, as correctly perceived by GPs participating in the study (54% of correct answers). Instead, poor knowledge of the underlying pathophysiological mechanisms seems to emerge, with only 46% of respondents correctly answering to the question on apnea/hypopnea in OSAHS). On the other side, most of the GPs perceive the importance of the association between OSAHS and obesity (71%) and between OSAHS and hypertension (63% of respondents), although in the latter case, the true prevalence is underestimated (Figure 3A). No GPs has identified the true prevalence of hypertension, which is around 50-60% in patients with a diagnosis of OSAHS. OSAHS as an independent cardiovascular risk factor was correctly identified by 84% of respondents (Figure 3B). Currently the knowledge of the questionnaire *“Sleepiness Epworth Scale”* is still poor. The latter assesses the likelihood of falling asleep or dozing off in various situations, which is a useful metric to suspecting OSAHS. 78% of the GPs surveyed did not know about it, while only 28% indicated to be aware but not to use it (Figure 4A). 60% of the GPs identifies the pulmonologist as the main contact person in the diagnosis of the OSAHS. 20% also identifies the need for a multidisciplinary team in the management of clinical and instrumental problem. The primary obstacles in accessing to specialist care were found to be the long waiting times (50%), the difficulty of convincing the patient (22%) and the complex interaction with the specialist (16%) (Figure 4B), emphasizing a lack of hospital-community collaboration that is rather fundamental in the clinical management of chronic disorders such as OSAS. 70% of respondents showed a good expertise in the diagnostic-therapeutic course of OSAHS, identifying in the polysomnography (Figure 5A) and in the C-PAP (Figure 5B) the diagnostic and therapeutic gold standards, respectively. 65% of GPs participating in the study is, however, aware of the lack of acceptance of the C-PAP by the patient, emphasizing the need for adequately trained personnel in education to the patient for the proper functioning of the C-PAP. In the 62% the respiratory therapist and the nurse are the healthcare providers who should compete primarily on the role of educator, while only 39% identified the GP and the pulmonologist for this role.

## Discussion and conclusions

OSAHS is a common disorder in the general population but often underestimated and underdiagnosed. OSAHS is an independent risk factor for hypertension and cardiovascular disease and is the frequent underlying disease of secondary hypertension and resistant hypertension [9]. Randomized, controlled trials have evaluated the use of continuous positive airway pressure (CPAP) to reduce BP among persons with OSA. The benefits of OSA treatment are related to implications for hypertension management. Several lines of evidence have also identified OSAHS as an independent risk factor of secondary hypertension, such as obesity. According to most studies, the treatment of obstructive breathing disorders during sleep makes a significant clinical improvement by reducing both blood pressure nocturnal diurnal [10]. The pathophy-siological mechanisms underlying this phenomenon are not yet clear. However, several mechanisms have been proposed, including increased sympathetic activity in response to hypoxemia and hypercapnia resul-ting in the chemoreceptor activation and increased peripheral vascular tone. Other processes implycated include vasoconstrictors in the circulation, humoral factors such as Endothelin-1 (ET-1) and endothelial dysfunction [11,12]. Furthermore, patients with OSAHS could be more easily predisposed to a weight gain. The simultaneous presence of obesity and OSAHS increases the risk of hypertension. In this study, the point of view of GPs showed a good knowledge of the OSAHS regarding the epidemiology, the associated risk factors, the diagnostic flow-chart, and therapeutic approach. The OSAHS is recognized by most of the GPs interviewed as disease in constant increase, associated with predisposing factors such as obesity, male sex, older age, use of hypnotics drugs and bad life habits such as alcohol abuse and cigarette smoking. High blood pressure is perceived as an independent cardiovascular risk factor in patients with OSAS, in line with major international studies and highlighting the importance of an appropriate treatment that can reduce blood pressure in both daytime and nighttime in these patients. Instead, the degree of knowledge of rating scales in the outpatients, such as Epworth Sleepiness Scale, resulted insufficient. The C-PAP has been identified as the gold standard of care in patients with OSAS, despite the lack of patient compliance in relation to this procedure, while polysomnography was found to be the main test instrument used in the diagnostic workup of OSAS. The pulmonologist and a multidisciplinary team have been identified as the specialist figures of reference to which to direct the patient through the diagnostic workup of OSAS despite the waiting time, the patient’s belief tiring and difficult interaction with the specialist frequently represent the major barriers in accessing to specialist care. Finally, respiratory therapists and nurses represent the role of educator in the proper management of the C-PAP in the opinion of 62% of respondents, while only 34% think that this role should be played by the GP and the specialist reference. Overall, it appears essential, as well as in all chronic diseases in the growing number of patients with adult-age, a proper understanding of the problem and an integrated hospital-community to guide the patient with OSAS through the proper procedures and best diagnostic and therapeutic able to prevent, or limit, the complications of this disease [13,14]. The results of this study would allow to take appropriate educational actions and this questionnaire could be used to monitor the possible effects of divulgation and educational initiatives over time.
